# The pannexins: past and present

**DOI:** 10.3389/fphys.2014.00058

**Published:** 2014-02-19

**Authors:** Stephen R. Bond, Christian C. Naus

**Affiliations:** ^1^Genome Technology Branch, Division of Intramural Research, National Human Genome Research Institute, National Institutes of HealthBethesda, MD, USA; ^2^Department of Cellular and Physiological Science, Life Sciences Institute, University of British ColumbiaVancouver, BC, Canada

**Keywords:** pannexin, Panx1, Panx2, Panx3, distribution, biochemistry, structure, gating

## Abstract

The pannexins (Panxs) are a family of chordate proteins homologous to the invertebrate gap junction forming proteins named innexins. Three distinct Panx paralogs (Panx1, Panx2, and Panx3) are shared among the major vertebrate phyla, but they appear to have suppressed (or even lost) their ability to directly couple adjacent cells. Connecting the intracellular and extracellular compartments is now widely accepted as Panx's primary function, facilitating the passive movement of ions and small molecules along electrochemical gradients. The tissue distribution of the Panxs ranges from pervasive to very restricted, depending on the paralog, and are often cell type-specific and/or developmentally regulated within any given tissue. In recent years, Panxs have been implicated in an assortment of physiological and pathophysiological processes, particularly with respect to ATP signaling and inflammation, and they are now considered to be a major player in extracellular purinergic communication. The following is a comprehensive review of the Panx literature, exploring the historical events leading up to their discovery, outlining our current understanding of their biochemistry, and describing the importance of these proteins in health and disease.

## Introduction

In 1959, Edwin Furshpan and David Potter observed action potentials propagating through the crayfish giant motor synapse with a delay of 0.1 ms, or about an order of magnitude faster than synaptic delays previously recorded. They also demonstrated that hyperpolarization of the post-synaptic fiber could “leak” backwards into the pre-synaptic fiber (Furshpan and Potter, 1959). These observations did not adhere to the canonical view of the synapse, whereby action potentials were understood to propagate unidirectionally via chemical intermediaries (Dale et al., 1936). The authors postulated a “synaptic rectifier” directly linking the cells, leading to the eventual discovery of intercellular gap junctions. These large aqueous pores span the plasma membranes of adjacent cells, creating a cytoplasmic syncytium through which ions and small molecules can rapidly diffuse. There are two large families of unrelated (yet structurally convergent) transmembrane proteins within the Metazoa that oligomerize into gap junctions, and these have been named the *connexins* (Cxs) and *innexins* (Inxs) (Panchin, 2005). The Cxs are found exclusively in chordates and have extensive physiological relevance; not only do Cxs act as junctional pipelines between cells, but their cytoplasmic components are also the site of numerous protein-protein interactions (Harris and Locke, 2008). Inxs appear to have evolved during the early days of animal multicellularity, so they are found throughout almost all branches of the Metazoa, and they too influence a diverse array of physiological processes. The following review will focus heavily on what is currently known about the chordate members of the Inx superfamily, which are more commonly known as *pannexins* (Panxs).

## Innexins

Following the original biophysical recording of gap junctions between arthropod neurons (Furshpan and Potter, 1959), subsequent studies found electrically equivalent features in other diverse metazoans, including the earthworm (Wilson, 1961), leech (Eckert, 1963), fish (Bennett et al., 1963), and mouse (Penn, 1966). The first Cx cDNAs were cloned in 1986 from mammalian liver samples (Kumar and Gilula, 1986; Paul, 1986) and, once identified, they were assumed to be the sole gap junction forming proteins, conserved throughout the animal kingdom. This assumption proved to be incorrect, however, as Cxs are actually exclusive to the chordate lineage (Panchin, 2005). The Inxs are a completely unrelated family of proteins responsible for forming gap junctions within the myriad invertebrata, with surprisingly similar functional and structural properties to the Cxs given their ancestral independence.

### Discovery

Throughout the decade following the first identification of mammalian Cx genes, homologous invertebrate genes remained strangely elusive. In 1994, Thomas Barns proposed that a small collection of genes present in the worm and fly might represent a new class of gap junctions; while they lacked any discernable sequence similarity to the Cxs, they did code for proteins with predicted structural similarities (Barnes, 1994). He named the group “OPUS” as an acronym for the genes *ogre*, *Pas*, *unc-7*, and *shakB*, which were those identified in his study. When *shakB* was later exogenously expressed in paired oocytes, it was, indeed, shown to encode a gap junction protein (Phelan et al., 1998b). The OPUS nomenclature was subsequently discarded as confusing because *Pas* and *shakB* are different alleles of the same gene, and “OPUS” fails to impart any sense of function to the group. The name of these genes was instead replaced with “invertebrate analogs of the connexins”—or, more simply, *innexins* (Phelan et al., 1998a). In the course of proposing this new name, Pauline Phelan mused over the fact that more than 90% of the *C. elegans* genome had been analyzed with no obvious sign of a Cx sequence (Phelan et al., 1998a). She postulated that the sequences were probably not present at all, and so the hunt for Cxs in worms and flies quietly came to an end. In the intervening years, the volume of genome sequencing data has expanded exponentially, and still there is no definitive sign of connexins outside the phylum Chordata, so it seems quite likely that if the Cx and Inx families are derived from an ancient gene duplication, their sequences have since diverged beyond our ability to infer that homology (Yen and Saier, 2007; Abascal and Zardoya, 2013). There are eight Inx genes in the genome of the arthropod *D. melanogaster* (Stebbings et al., 2002; Bauer et al., 2005), 25 in the nematode *C. elegans* (Altun et al., 2009), and 21 in the annelid *H. verbana* (Kandarian et al., 2012), with many more identified throughout the various metazoan clades (Yen and Saier, 2007). The protein products from all of these genes are clearly homologous, apparently originating from a single gene that would have been present in the ancestral species at the base of the Metazoa some 600 million years ago (Yen and Saier, 2007).

### Structure and function

Inx proteins are predicted to have four transmembrane domains, one intracellular and two extracellular loops, and cytoplasmic C- and N-terminal tails (Barnes, 1994). There are two highly conserved cysteine residues in both of the extracellular loops (Yen and Saier, 2007), which are likely under positive selection for proper channel formation and function (Bunse et al., 2011). Inx monomers are thought to oligomerize into hexameric pore structures (known as “innexons”) that loosely aggregate at points of very close cell-to-cell contact (Tazuke et al., 2002; Lehmann et al., 2006; Oshima et al., 2013). This may allow them to behave in an analogous fashion to Cxs, with a hexamer from one cell “docking” with another on the adjacent cell, thus forming intercellular gap junctions (Phelan et al., 1998a). Innexons can be constructed from a single (“homomeric”) isotype or multiple (“heteromeric”) isotypes (Stebbings et al., 2000), and docking between innexons can be “homotypic” or “heterotypic,” depending on whether each half is constructed from the same isotype or not (Wu et al., 2011). In terms of pore activity, representative Inxs from fly, nematode, and leech have been shown to form highly conductive, non-specific, transjunctional voltage-sensitive intercellular channels typical of a chordate gap junction (Phelan et al., 1998a; Landesman et al., 1999; Stebbings et al., 2000; Bao et al., 2007; DePriest et al., 2011). Several properties set this group apart, however, from what is currently known of Cx-based channels. For example, transjunctional voltage (V_*j*_) is understood to have a gating effect on Cx based channels irrespective of the isotype involved (González et al., 2007), while gap junctions consisting of *H. medicinalis* inx1 (leech) are completely insensitive to V_*j*_ (Dykes et al., 2004); *D. melanogaster* inx2 is dependent upon inx3 as a carrier for proper localization to the plasma membrane (Lehmann et al., 2006); and, although not yet shown conclusively, it is thought that some of the Inxs in the *C. elegans* reproductive system do not create gap junctions at all, perhaps in favor of forming functional innexons called “hemichannels” (Altun et al., 2009). Very few Inx structural features have been explored experimentally, but tryptophan scanning of *D. melanogaster* inx8 has recently shown residues H27, T31, L35, and S39 (all in the first transmembrane domain) to be positioned along one face of an α-helix that is likely involved in a helix-helix interaction necessary for channel function (DePriest et al., 2011). Further, negative stain electron microscopy indicates that the *C. elegans* INX-6 channel has a diameter of ~140 Å (Oshima et al., 2013).

Broad surveys of Inxs in *D. melanogaster* and *C. elegans* show complex expression patterns throughout embryogenesis and in adult tissues, often exhibiting isotype overlap (Stebbings et al., 2002; Altun et al., 2009). Disrupting these normal expression patterns can have harmful consequences; for instance, *Inx7* is necessary for proper axon guidance in the peripheral nervous system of the *D. melanogaster* embryo (Ostrowski et al., 2008), and in conjunction with *Inx6*, is also required for long term memory formation (Wu et al., 2011). In the worm, phenotypes associated with Inx disruption include the *eat-5*, and *unc-7*/*unc-9* mutants (three of the earliest observed, and the only *C. elegans* Inx genes to not conform to the standard naming scheme), which result in desynchronization of pharyngeal muscle contractions (Starich et al., 1996), and severe impairment of forward movement along with egg-retention, respectively (Starich et al., 1993; Barnes and Hekimi, 1997).

## Pannexins

As discussed above, the Cxs are chordate-specific, while invertebrates utilize Inxs to form intercellular gap junctions. It so happens that Inxs are also expressed alongside Cxs in chordates, but instead of competing with the Cxs as a redundant class of gap junction proteins, this family—collectively known as the *pannexins* (Panxs)—primarily oligomerize into distinct aqueous pores between the intracellular and extracellular space. Considerable attention has been focused on the biochemical and functional properties of Panxs since they were first reported in 2000, and much of the literature pertaining to this work will be reviewed here.

### Discovery

Yuri Panchin was the first to report how extensively the Inx genes have radiated throughout the metazoans by identifying homologs in disparate protostomal phyla (Platyhelminthes, Nematoda, Arthropoda, and Mollusca), as well as in the human genome (Panchin et al., 2000). In light of this Inx diversity, and especially because of their presence in non-invertebrates, Panchin argued that the existing naming convention was not appropriate. He suggested that this family of genes be re-branded as “pannexin,” because the Latin prefix “pan,” meaning “all,” would better represent the phylogenetic range in which they have been identified (Panchin et al., 2000). Unlike the switch from OPUS, the community was already becoming accustomed to the “Innexin” nomenclature, and it has remained largely unchanged when referring to invertebrate gap junction genes. Even so, the new name *has* been adopted in the case of chordate Inx homologs, and from lancelet to man these genes are known as pannexins.

The initial discovery of mammalian Panxs was achieved by performing BLASTP and PSI-BLAST searches against GenBank, using Inx sequences from several different invertebrate phyla as the query (Panchin et al., 2000); subsequent studies have since applied further statistical methods to confirm homology between Inxs and Panxs (Baranova et al., 2004; Phelan, 2005; Yen and Saier, 2007; Fushiki et al., 2010). As with the Inx genes from worm and fly, significant conservation of primary amino acid sequence is not seen between the Cxs and Panxs, despite having similar structural topologies (Fushiki et al., 2010). There has, however, been a recent discovery of another protein that may share ancestry with Panxs—specifically, the leucine-rich repeat containing 8 (LRRC8). It appears that the entire transmembrane region of an ancient Panx gene may have fused with a leucine-rich repeat (LRR)-containing domain, with the LRR taking on the role of a new C-terminal tail (Abascal and Zardoya, 2012). To date, there have been no biophysical analyses of this unique protein, but if it represents yet another large plasma membrane channel, it could explain some of the conflicting reports regarding permeability within the Panx literature that are discussed in subsequent sections of this review.

### Biochemical properties

Panxs form low-resistance channels at the plasma membrane, with multiple distinct gating mechanisms. Post-translational modifications to the monomeric peptides are critical for proper intracellular localization and channel function, and a rapidly growing list of interacting proteins implies a complex and multifunctional role for Panxs in cellular processes.

#### Isotypes

Vertebrate Panx genes have been grouped into three broad isotypes, and the products of these genes have been designated Panx1, Panx2, and Panx3. Duplication events that occurred prior to the radiation of the subphylum Vertebrata are responsible for creating these Panx paralogs and, as a result, all three are orthologous across taxa [e.g., Panx2 in *Xenopus* is equivalent to Panx2 in human (Abascal and Zardoya, 2013)]. Sequence alignments indicate that Panx orthologs share an average of >70% identity and >80% similarity at the amino acid level (Table 1). Panx1 and Panx3 are also relatively well-conserved, sharing ~60% identity and ~75% similarity between paralogs, but Panx2 is significantly more divergent (Table 2). Several splice variants have been predicted for Panx1 and Panx2, with experimental evidence for at least three different Panx1 isoforms in rat (Baranova et al., 2004; Li et al., 2011b; Turmel et al., 2011), and at least two Panx2 isoforms in fish (Zoidl et al., 2008). The teleost fishes also have a fourth Panx paralog, retained as two “ohnologs” of Panx1 following a whole genome duplication event ~350 million years ago (Bond et al., 2012). This duplication presents an interesting opportunity to assess the plasticity of Panx1 when evolutionary pressures are relaxed; indeed, the two paralogs are already known to have diverged in terms of both transcriptional regulation and channel activity (Bond et al., 2012; Kurtenbach et al., 2013).

**Table 1:** **Amino acid conservation of Panx orthologs**.

	**Panx1**	**Panx2**	**Panx3**
Identity	73.5 ± 3.0%	71.3 ± 4.9%	75.5 ± 5.9%
Similarity	82.6 ± 3.0%	80.0 ± 4.4%	85.2 ± 4.4%

*To calculate percent conservation of a given ortholog pair (e.g., between mouse and chicken Panx1), a pairwise global alignment was first constructed. “Identity” is defined as an exact match between two aligned residues, and “similarity” indicates positions in an alignment where conservative substitutions are observed (i.e., a score of greater than zero using the BLOSUM45 substitution matrix). The values presented represent average conservation (plus or minus one standard deviation) of all possible combinatorial alignments among Panx orthologs from M. musculus, G. gallus, X. tropicalis, L. chalumnae, and D. rerio*.

**Table 2:** **Amino acid conservation of Panx paralogs**.

	**Panx1/Panx2**	**Panx1/Panx3**	**Panx2/Panx3**
Identity	24.9 ± 2.5%	62.4 ± 3.2%	25.9 ± 1.9%
Similarity	37.0 ± 2.0%	74.2 ± 3.0%	37.0 ± 2.6%

*To calculate percent conservation of a given paralog pair (e.g., between mouse Panx1 and mouse Panx2), a pairwise global alignment was first constructed. “Identity” is defined as an exact match between two aligned residues, and “similarity” indicates positions in an alignment where conservative substitutions are observed (i.e., a score of greater than zero using the BLOSUM45 substitution matrix). The values presented represent average conservation (plus or minus one standard deviation) of all possible combinatorial alignments among Panx paralogs from M. musculus, G. gallus, X. tropicalis, L. chalumnae, and D. rerio*.

#### Structure

**Figure 1:**
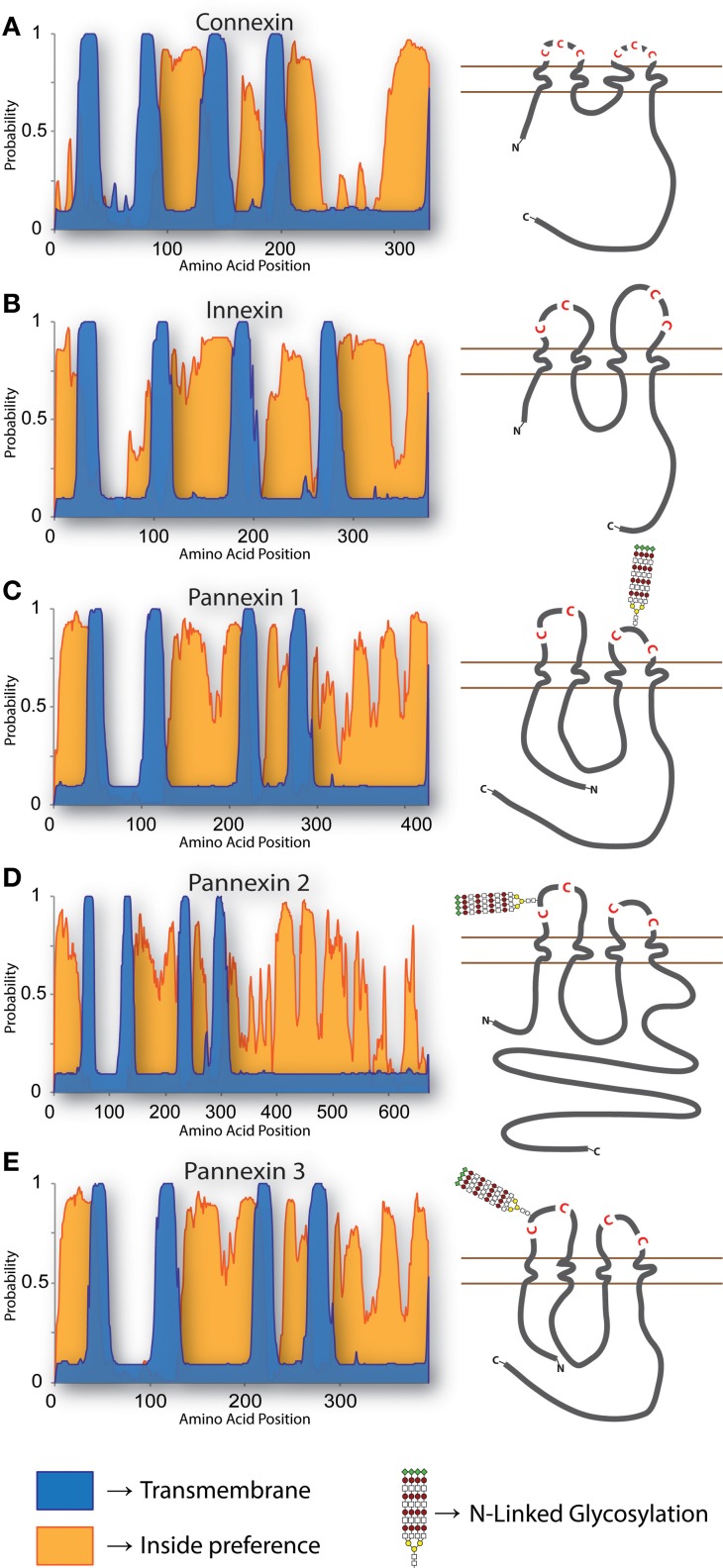
**Topology of Cxs, Inxs, and Panxs**. The predicted arrangement of these proteins within a membrane has been reported on multiple occasions (Hua et al., 2003; Panchin, 2005; Barbe et al., 2006; Magie and Martindale, 2008). To generate the predictions shown here, multiple pairwise alignments were calculated with the E-INS-i algorithm of MAFFT (Katoh and Standley, 2013) using **(A)** all 21 human Cxs, **(B)** 25 *C. elegans* Inxs, and **(C–E)** the three Chordate Panx isotypes using protein sequences from mouse, chicken, *Xenopus*, coelacanth, and *Danio*. Transmembrane (TM) regions and ER integration orientation were predicted with the OCTOPUS algorithm (Viklund and Elofsson, 2008), using the consensus sequences from each multiple alignment as input. All of the proteins have compelling support for 4 TM domains (blue), while the “inside-preference” (orange; based on clustering of positively charged and polar residues) assigned to the N-terminus and second loop strongly suggests that the termini are cytoplasmic. The topologies of each consensus sequence are shown to scale, with relative positions of extracellular loop cysteines and glycosylated asparagines indicated.

Secondary structure and hydrophobicity prediction algorithms indicate that the gross topology of Panx conforms to that of the Cx and Inx superfamilies, which includes four transmembrane domains with cytoplasmic C- and N-termini (Figure 1). Site-directed mutagenesis experiments have demonstrated that four conserved extracellular cysteine residues are required for functional Panx1 channel formation (Ambrosi et al., 2010; Wang and Dahl, 2010; Bunse et al., 2011). Of the other conserved Panx1 cysteines, converting the intracellular residue C346 or the transmembrane residue C40 to serine results in a constitutively open channel that rapidly degrades plasma membrane potentials, leading to cell death (Bunse et al., 2010, 2011; Wang and Dahl, 2010). On the other hand, replacing the same residues with hydrophobic alanine is non-toxic to transfected cells, so this substitution probably does not result in a constitutively open channel (Lohman et al., 2012b). Feng Qiu systematically modified 81 of the 84 extracellular Panx1 residues to alanine, one at a time, followed by careful electrophysiological characterization of the mutant proteins' ability to form channels (Qiu et al., 2012). Of the mutants created, 24 ablated voltage gated channel activity; how each mutation altered channel activity, be it through changes in folding, trafficking, or direct channel blockage, was outside the scope of that particular report however. To gain a better understanding of the actual Panx1 pore structure, the substituted-cysteine accessibility method and electron microscopy have both been used, and it appears that residues 3–7, 10, and 12 from the N-terminus, residues 58–62 from the first transmembrane domain, and residues 414–426 from the extreme C-terminus constitute the hydrophilic pore lining moieties (Wang and Dahl, 2010), with the outer entrance of the pore estimated at a diameter of ~17–21 Å (~29.5–30.5 Å in the case of Panx2) (Ambrosi et al., 2010).

#### Oligomerization

Panxs were shown to form channels very soon after they were first discovered, so it was assumed that the monomeric protein oligomerized into a hexameric structure analogous to the connexon (Bruzzone et al., 2003). Electron microscopy of cell membranes expressing Panx1 or Panx2 supports this notion of a pannexon (Ambrosi et al., 2010), as do cross-linking experiments where SDS-PAGE and Western blots reveal Panx1 bands at sizes expected for dimeric and hexameric interactions (Boassa et al., 2007). Intriguingly, similar cross-linking experiments for Panx2 suggest that they may actually form octameric structures, which is a arrangement that Cxs have not been reported to assume (Ambrosi et al., 2010). Cross-linking experiments have not been performed using Panx3, which has an approximate monomeric weight of 42 kDa, but a Panx3 band with a mass of about 70 kDa has been observed in standard Western blot experiments (Penuela et al., 2007). This larger band was initially thought to represent a dimer resistant to dissociation during SDS-PAGE (Penuela et al., 2007; Celetti et al., 2010; Turmel et al., 2011), but further efforts to disrupt it with stronger reducing agents have been unsuccessful; the most recent hypothesis is the existence of an alternative Panx3 splice variant (Cowan et al., 2012). We too have observed a strong 70 kDa Western blot band in lysates taken from cultured cells, but we have also identified a similarly sized band in fetal bovine serum (FBS) (Bond, 2012). It is currently unclear whether the FBS band represents the same glycoprotein that Cowan et al. (2012) visualized using three different antibodies, but investigators should be cognizant of FBS as a source of experimental error when interpreting their results.

Immunoprecipitation experiments have suggested that heteromeric pannexons can also form, composed of Panx1 and Panx2 or Panx1 and Panx3, but not between Panx2 and Panx3 (Bruzzone et al., 2005; Penuela et al., 2009). However, heteromeric intermixing does not appear to be conducive to channel activity, with Panx1/2 co-expression reducing the size of voltage-activated currents relative to Panx1 expression alone (Bruzzone et al., 2005). The configuration is also not overly stable, as Panx1/2 pannexons degrade much more rapidly than their monomeric counterparts (Ambrosi et al., 2010).

#### Posttranslational modification

Phosphorylation is such a crucial regulator of Cx function and life cycle (Solan and Lampe, 2009; Chen et al., 2012) that it was surprising when phosphatase treatment failed to cause a mobility shift of Panx during SDS-PAGE (Boassa et al., 2007; Penuela et al., 2007). More recently, however, indirect evidence for Src family kinase phosphorylation of tyrosine 308 of Panx1 has emerged, quite possibly as a means of opening the channel (Weilinger et al., 2012). Furthermore, broad specificity anti-phospho antibodies suggest that the threonine residue at position 387 and one or more of the serine residues at positions 159, 206, 328, 343, 394, and 405 of Panx1 can be phosphorylated (Riquelme et al., 2013). As was reported for tyrosine 308 phosphorylation, serine/threonine phosphorylation is also correlated with increased membrane permeability.

The addition of S-nitrosothiol groups onto cysteine residues is a broadly utilized form of reversible protein modification, and both Panx1 and Panx3 are receptive to exogenous application of nitric oxide (NO) donor molecules (Lohman et al., 2012b; Penuela et al., 2014). S-nitrosylation of Panx1 residues 40 (first transmembrane domain) and 346 (C-terminal tail) closes the channel (Lohman et al., 2012b), providing a tantalizing model for the regulation of Panx1 activity in the circulatory system where NO is an important paracrine factor. It is still unclear, however, whether this is an important mechanism *in vivo*. Panx2 is not susceptible to S-nitrosylation (Penuela et al., 2014), but it may be a target for palmitoylation on residue 246, where it has been suggested to regulate membrane targeting (Swayne et al., 2010).

While thorough analyses into how phosphorylation, nitrosylation, and palmitoylation affect Panx dynamics are only just beginning, glycosylation is a well-described feature. Sugar moieties are first attached in the endoplasmic reticulum (ER) to residue N254 of Panx1 (N246 on both fish ohnologs, plus N71 and N95 on Panx1b), N86 of Panx2, and N71 of Panx3 to create high mannose chains (Dvoriantchikova et al., 2006a; Boassa et al., 2007; Penuela et al., 2007, 2009; Prochnow et al., 2009b; Kurtenbach et al., 2013). Panx1 and Panx3 then move into the Golgi for further processing of their high mannose groups into a mature “complex” form before they move on to the plasma membrane, while the sugars attached to Panx2 appear to stay as high mannose (Penuela et al., 2009; Bhalla-Gehi et al., 2010). Blocking N-glycosylation through site directed mutagenesis or by pharmacological agents reduces the ability of Panxs to traffic to the plasma membrane (Boassa et al., 2007; Penuela et al., 2007; Kurtenbach et al., 2013). Neither sialylation nor O-glycosylation appear to be a feature of any Panx isotype (Penuela et al., 2014).

#### Interacting proteins

Co-immunoprecipitation has been the predominant method used to identify the growing list of Panx-interacting proteins. As will be discussed in greater detail in section Gating, there is a well-established functional relationship between Panx1 and the ATP-gated ion channels known as P2X receptors, but there is also a physical relationship. Of the seven P2X receptor subtypes, P2X_2_R, P2X_3_R, P2X_4_R, and P2X_7_R have been shown to co-immunoprecipitate with Panx1 (Pelegrin and Surprenant, 2006; Silverman et al., 2009; Li et al., 2011b; Poornima et al., 2012; Hung et al., 2013; Kanjanamekanant et al., 2013), and in the case of P2X_7_R, proline 451 within an SH_3_ domain of the C-terminal tail of the receptor is involved in the interaction (Iglesias et al., 2008; Sorge et al., 2012). P2X_7_R helps regulate innate immunity through an association with a multi-protein complex known as the inflammasome, and Panx1 also co-immunoprecipitates with many inflammasome components, including NLRP1, NLRP2, NLRP3, ASC, caspase-1, caspase-11, and XIAP (de Rivero Vaccari et al., 2009; Silverman et al., 2009; Minkiewicz et al., 2013; Wang et al., 2013a). It still remains to be determined whether these proteins bind directly to Panx1 or if the associations are adaptor-mediated. G protein-coupled P2Y receptors are a second major class of ATP binding proteins that are thought to associate with Panx1, probably as part of a complex (Buvinic et al., 2009).

The C-tail of Panx1 co-immunoprecipitates with actin and the actin interacting protein Arp3 (Bhalla-Gehi et al., 2010; Wicki-Stordeur and Swayne, 2013), and pharmacological disruption of the microfilaments (but not of tubulin microtubules) reduces cell surface stability and motility of both Panx1 and Panx3 (Bhalla-Gehi et al., 2010). In line with a direct interaction between the actin cytoskeleton and Panx, there is significant localization of zebrafish Panx1 to active areas of the cell membrane where actin is highly dynamic, such as ruffles (Bond et al., 2012).

Other proteins that have been shown to associate with Panxs include Kvβ 3, which regulates the gating effects of redox potential on Panx1 (Bunse et al., 2005, 2009); the α1-adrenergic receptor, possibly as part of a signaling microdomain that controls vascular smooth muscle tone (Billaud et al., 2011); a voltage-gated L-type calcium channel, Cav1.1 (also known as the dihydropyridine receptor), which is found in the transverse tubules (T-tubules) of skeletal muscle and might play a role in mediating an interaction between Panx1 and P2Y_2_ (Buvinic et al., 2009; Jorquera et al., 2013; Valladares et al., 2013); SAP97, which facilitates protein complex formation and trafficking to the plasma membrane via an association between an SH_3_-Hook-guanylate kinase (GUK) domain and the C-terminal tail of Panx1 (Dolmatova et al., 2012); and stomatin, which inhibits Panx1 channel activity when bound to the C-terminus (Zhan et al., 2012).

### The pannexin channel

Given their relationship to the Inxs, it was initially thought that Panxs were gap junction-forming proteins. Today, that notion has mostly been repudiated, and Panxs are now understood to form large gated pores between the intracellular and extracellular space, in a way that is reminiscent of Cx hemichannels.

#### Intercellular gap junctions

A small body of work indicates that, under certain circumstances, Panx-based intercellular channels will form. For example, over-expression in *Xenopus* paired oocytes reveals appreciable levels of transjunctional current attributable to Panx1 or co-expressed Panx1/2 (Bruzzone et al., 2003). However, it takes a very long time for these channels to accumulate (upwards of 24 h), and the macroscopic currents are weak compared to Cx-based gap junctions (Boassa et al., 2007). For gap junctions, these channels are uncharacteristically insensitive to transjunctional voltage, with recorded currents varying linearly with membrane potential even at large driving forces (> +60 mV or < −60 mV) (Bruzzone et al., 2003); with Cx-based gap junctions, differences in electrical potential between coupled cells inactivates the channel (González et al., 2007).

Intriguingly, enzymatic removal of bulky N-glycans from the cell surface dramatically enhances the ability of Panx1 pannexons to couple (Boassa et al., 2008), implying that even though the Panxs may retain an ancestral ability to form patent gap junctions, this configuration is largely inhibited *in vivo* through steric interference. Nonetheless, Panx gap junctions have still been inferred following over-expression in cultured cell lines. Rat C6 glioma cells appear to acquire the ability (albeit a weak one) to pass sulforhodamine 101 when Panx1 is introduced (Lai et al., 2007), and the human prostate adenocarcinoma line LNCaP shows increased movement of calcium between neighboring cells following Panx1 transfection (Vanden Abeele et al., 2006). Panx2 has not been reported to form gap junctions, and the only evidence for Panx3-based intercellular channels is dependent on measurements of calcium wave propagation between cultured osteoblast (Ishikawa et al., 2011).

Concerns and rebuttals have been published in response to all of the studies supporting Panx-based coupling, such as the potential for a non-physiologically relevant response to massive over-expression, a lack of proper control for induction of endogenous Cxs, and a general inability within the community to observe Panx behaving in a fashion expected from a gap junction, such as sequestration to areas of cell-to-cell contact (Dahl and Harris, 2009; Ambrosi et al., 2010; Sosinsky et al., 2011; Penuela et al., 2012). In reality, the general attitude toward Panx gap junctions has largely degraded from “common cellular feature” to that of “academic peculiarity.” The hypothesis that the primary function of Panx is to form non-junctional channels was first formally outlined in 2006 (Dahl and Locovei, 2006), and while a complete disavowal of physiologically relevant Panx gap junctions may not necessarily be prudent, the burden of proof currently rests heavy on the shoulders of anyone making such a claim.

#### Non-junctional channels

Since the very first biophysical study performed by Roberto Bruzzone in 2003, Panx1 has been known to form non-junctional channels that mediate robust macroscopic currents across the plasma membrane of individual cells (Bruzzone et al., 2003). These channels have often been characterized by a unitary conductance ranging from ~300 pS to >500 pS, depending on the charge carrier (Bao et al., 2004; Locovei et al., 2006a; Prochnow et al., 2009a,b; Iglesias et al., 2010; Kurtenbach et al., 2013), with non-selective permeability to solutes smaller than 1.0–1.5 kDa (Wang et al., 2007). The channel tends to open at positive membrane potentials and requires 30–70 ms to transition from fully closed to fully open (Bruzzone et al., 2003; Bao et al., 2004; Ma et al., 2009; Prochnow et al., 2009b). There are also at least five open substates (corresponding to 5, 25, 30, and 90% of the fully open unitary conductance) (Bao et al., 2004) and, following activation, Panx1 channels will often deactivate again over several seconds to a lower subconductance state (Bruzzone et al., 2003; Bao et al., 2004). It is becoming clear, however, that the cellular context in which Panx finds itself has influence over the channel's biophysical properties, as recent unitary recordings of Panx1 channels excised from over-expressing HEK293 cells reveal an upper conductance limit of 60–70 pS (Romanov et al., 2011, 2012; Ma et al., 2012) and size exclusion of molecules as small as 237 Da (Romanov et al., 2012). While studying the zebrafish Panx1b-EGFP channel, over-expressed in HeLa cells, we too have observed a much smaller unitary conductance (123 pS) from excised membrane patches (Bond et al., 2012). Cell type must be carefully chosen when designing future experiments, and investigators should be especially mindful of the conditions used during recordings if attempting to compare between their own results and those reported in other studies. To help the field reach consensus, a standardized patch clamp paradigm for eliciting Panx1 currents has been proposed by Grundken and colleagues, so if practical, investigators may want to consider adopting their method (Grundken et al., 2011).

The initial oocyte studies, which revealed such robust Panx1 channel activity in response to membrane potential, completely failed to note currents attributable to either Panx2 or Panx3 (Bruzzone et al., 2003). Subsequent work using sulforhodamine B uptake as a readout suggests that both of these proteins can indeed form cell surface channels that are activated by mechanical stimulation (i.e., from the fluid shear force caused by dripping dye directly onto monolayer cell culture) (Penuela et al., 2007, 2009; Celetti et al., 2010). Other work has taken the analysis of Panx2 a step further by showing how purified baculovirus liposomes become permeable to ascorbate if Panx2 is present, and how electrophysiological measurements are possible in oocytes if a protracted voltage ramp protocol (−100 to +100 mV over 70 s) is implemented (Ambrosi et al., 2010). Heteromeric Panx1/2 pannexons also show channel activity, but the resultant currents are significantly lower than those from homomeric Panx1 channels, and no channel activity has been reported for Panx1/3 or Panx2/3 heteromers (Bruzzone et al., 2003).

#### Pharmacological blockers

To help tease apart the details of Panx function, efficacious and highly selective drugs that block channel activity are desirable. Several compounds have been identified that suppress Panx1 currents, with varying potency and specificity, and some have been used to great effect in the collective effort to understand the physiological relevance of this channel. Carbenoxolone (CBX) is a synthetic derivative of glycyrrhetinic acid (found in licorice) that has been used to reversibly block Cx-based channels for over 25 years (Davidson et al., 1986). CBX was also one of the first agents shown to directly inhibit Panx1 currents (Bruzzone et al., 2005), although Panx channels are much more sensitive to the drug than are Cx channels (Bruzzone et al., 2005), so low-dose CBX (10–30 μ M) is commonly used to differentiate between Panx activity and Cx hemichannels (Huang et al., 2007; Ma et al., 2009; Li et al., 2012a; Zhang et al., 2012b). Mefloquine is another compound that reversibly blocks both Panx and Cx channels at different concentrations; Panx1 channels are inhibited with an IC_50_ of ~50 nM (Iglesias et al., 2010), as compared to >10 μ M for many Cx isotypes (Cruikshank et al., 2004). A note of caution when using mefloquine, however: there are marked differences in the efficacy of the two common diastereomers, and certain commercial sources may be much less potent than others (Iglesias et al., 2010). The drug probenecid (clinically used to treat the symptoms of gout) has proven popular (Dando and Roper, 2009; Lamkanfi et al., 2009; Ransford et al., 2009; Billaud et al., 2011; Xia et al., 2012) after it was shown to block Panx1 channels with an IC_50_ of ~150 μ M without affecting Cx channels, even at doses as high as 5 mM (Silverman et al., 2008). Interestingly, the effects of probenecid and CBX on Panx1 are severely mitigated if the potassium channel subunit Kvbeta3 is present, so in studies where these blockers fail to have an effect, the expression of Kvbeta3 should be assessed before ruling out Panx1 activity (Bunse et al., 2009). The chloride channel blocker 5-nitro-2-(3-phenylpropylamino)benzoic acid (NPPB) blocks Panx1 activity with an IC_50_ of ~50 μ M but, unfortunately, it also acts on Cx hemichannels at a similar concentration (Silverman et al., 2008). Another popular reagent is a mimetic peptide against the first extracellular loop of Panx1, called ^10^Panx1, which impedes passage of small ion currents, dyes, and ATP (Pelegrin and Surprenant, 2006, 2007; Wang et al., 2007; Reyes et al., 2009; Montalbetti et al., 2011). This effect can be partially replicated by other peptides of equivalent size, or by polyethylene glycol with a molecular weight of 1500 Da, so the effect may be caused by molecules of a certain size becoming lodged within the pore itself rather than from specific interactions with the extracellular loop (Dahl, 2007; Wang et al., 2007). Two compounds that show more recent promise are the anti-diabetic drug glyburide and the food additive Brilliant Blue FCF (BB FCF). Glyburide was originally developed to block ATP-regulated potassium channels, but it will also inhibit Panx1 activity with an IC_50_ of 45 μ M (Qiu et al., 2011). Glyburide's effect on Cx channels has not been reported. BB FCF inhibits Panx1 at an IC_50_ of 270 nM, with no measurable effect on Cx46 or Cx32E_1_43 hemichannels at concentrations as high as 100 μ M (Wang et al., 2013b).

As with all pharmacologically based experimentation, off-target effects must be considered when drawing conclusions from observations in the presence of Panx inhibitors. More stark attention has been drawn to this caveat with the recent availability of Panx knockout mice; for example, dramatic effects on the progression of chlamydia infection are seen in the presence of probenecid, CBX, and glyburide, yet infection and sensitivity to these compounds are the same for both wild-type and Panx1^−/−^ cells (McKuen et al., 2013). This is not an isolated case either, and more examples will be discussed in subsequent sections. All authors are strongly encouraged to implement as much redundancy as logistically possible when designing experiments that target Panx activity, using a minimum of two blocking agents with different modes of action, and preferably including a knock-down or knockout approach. For a more thorough discussion of Panx1 inhibitors, please refer to Dahl et al. (2013).

#### Gating

Panx1 channels are generally gated shut to prevent degradation of the electrochemical gradient across the plasma membrane. The channels are sensitive to voltage, and have very low open probability at the normal (i.e., negative) resting potential (Bruzzone et al., 2003). At positive voltages, the characteristic high conductance pore does become evident, but aside from excitable cell types like neurons and myocytes, membrane potentials above 0 mV are uncommon. Panx1 channel activity is also suppressed by acidification of the cytoplasm (Locovei et al., 2006b; Kurtenbach et al., 2013), increased intracellular redox potential (Bunse et al., 2009, 2010, 2011), S-nitrosylation (Lohman et al., 2012b), arachidonic acid (Samuels et al., 2013), and (at least partially) through an interaction with an erythrocyte integral membrane protein called stomatin (Zhan et al., 2012). To date, four distinct channel activation mechanisms have been identified:

1. Mechanical stimulation, like increased fluid shear force (Penuela et al., 2007, 2009; Celetti et al., 2010; Forsyth et al., 2011), the application of suction to a patch of excised membrane (Bao et al., 2004), or the physical stretching of entire cells (Xia et al., 2012) increases permeability for the duration of the stimulus. Cell swelling has also been proposed as a semi-mechanical stimulus for Panx1 activation (Locovei et al., 2006a; Li et al., 2010, 2011a,b, 2012a; Islam et al., 2012; Wicki-Stordeur and Swayne, 2012; Xia et al., 2012), although compelling arguments have been made that other channels are often at least partly responsible for solute release following hypotonic stress (Liu et al., 2008; Reyes et al., 2009; Islam et al., 2012).
2. Increasing extracellular potassium concentration ([K^+^]_*o*_) to ≥10 mM causes Panx1 channels to open even at hyperpolarized potentials as low as −100 mV (Silverman et al., 2009; Santiago et al., 2011; Suadicani et al., 2012). Enhanced Panx1 activity has also been observed at very high [K^+^]_*o*_, in the 100 mM range, but the resultant membrane depolarization in these experiments would also have a strong activating effect (Bao et al., 2004).
3. Proteolytic cleavage of the Panx1 C-terminal tail by caspase 3 or caspase 7 at residues 376–379 results in a constitutively open channel, generally in association with apoptosis (Chekeni et al., 2010; Qu et al., 2011). Non-covalent binding appears to occur between the C-tail and the pore interior, which dissociates once the C-tail has been cleaved (Sandilos et al., 2012). Interestingly, adding an exogenous peptide into the cell with the same sequence as the cleaved C-tail fragment is sufficient to block the processed channel, but only at negative membrane potentials. The peptide has no effect at positive potentials, so the cleaved channel behaves “normally” as long as the peptide is present in a high enough quantity to match its dissociation constant, thus implying that the extreme C-tail is involved in the overall voltage sensitivity of the Panx1 channel (Sandilos et al., 2012). Panx2 is also a substrate for caspase 3 and caspase 7, with cleavage occurring somewhere between residues 373 and 479 on the C-terminal tail (Penuela et al., 2014). Penuela et al. also report that Panx3 is not targeted by caspase, and the consequences of Panx2 cleavage remain to be explored.
4. Unlike the Cxs, Panxs are insensitive to extracellular calcium ([Ca^2+^]_*o*_) (Bruzzone et al., 2005; Ma et al., 2009). On the other-hand, rises in *intracellular* calcium ([Ca^2+^]_i_) to mM levels allow Panx1 channels to open readily at normal resting potentials (Locovei et al., 2006b; Prochnow et al., 2009b; Orellana et al., 2013a). Several physiological processes known to generate this level of [Ca^2+^]_i_ have been suggested or shown to influence Panx1 activity, including caffeine stimulation of the ryanodine receptor (Kienitz et al., 2011), thrombin activation of protease-activated receptor-1 or histamine activation of the histamine receptor (Godecke et al., 2012; Pinheiro et al., 2013b), adrenergic receptor alpha1 and muscarinic acetylcholine receptor activation of q-subtype G-protein (Sumi et al., 2010) and, perhaps most importantly, stimulation of various purinergic receptors (Locovei et al., 2006b; Pelegrin and Surprenant, 2006). Members from the P2Y and P2X receptor classes have both been implicated in ATP-mediated Panx1 activation, but each does so via different mechanisms. P2Y_1_R and P2Y_2_R are G protein-coupled receptors that mediate calcium release from internal stores through phospholipase C-dependent inositol 1,4,5-triphosphate (IP_3_) generation (Kügelgen, 2006), and both induce Panx1 currents following ATP stimulation (Locovei et al., 2006b; Zhang et al., 2012b). On the other hand, the P2XRs are themselves channel proteins and, upon activation, can increase [Ca^2+^]_i_ by facilitating transit across the plasma membrane from the extracellular space. Direct interaction between P2XRs and Panx1 (Li et al., 2011b) could ensure that local increases in [Ca^2+^]_i_ are detected by Panx channels; this would result in a biphasic current as ions first flow through open P2XRs, followed by a larger current as Panx1 channels open in response. ATP-dependent biphasic currents have in fact been measured, and they have also shown sensitivity to pharmacological inhibition and knockdown of Panx1 (Pelegrin and Surprenant, 2006; Iglesias et al., 2008), but extreme care must be taken. Large secondary pores have still been observed following ATP treatment when Panx1 is knocked-down or knocked-out, possibly due to P2X_7_R dilation or through an interaction with another as yet unidentified channel (Hanley et al., 2012; Rigato et al., 2012; Xu et al., 2012; Alberto et al., 2013). Furthermore, the P2X_7_R antagonists brilliant blue-G and KN-62 have both been reported to *increase* Panx1 channel function under normoxic and ischemic conditions in cultured rat astrocytes, suggesting that P2X_7_R might be *inhibitory* toward Panx1 under certain conditions (Iwabuchi and Kawahara, 2011). Unfortunately, the results of Iwabuchi and Kawahara are somewhat difficult to interpret, because the rat P2X_7_R ortholog is known to be completely insensitive to KN-62 (Donnelly-Roberts et al., 2009), and multiple independent sources have come to the opposite conclusions when employing these drugs (Iglesias et al., 2008; Qiu and Dahl, 2009; Lemaire et al., 2011).

A consequence of Panx1 activation by P2 receptors is the potential for positive feedback. With a pore permeable to solutes up to 1.5 kDa (Wang et al., 2007), Panx1 channels are expected to allow ATP (0.5 kDa) to flow along its concentration gradient from the cytosol to the extracellular space, where more cell surface purinergic receptors await activation. Indeed, the permeability of Panx1 channels to ATP was first demonstrated with a set of clever reversal potential measurements, using a patch pipette with an ATP gradient between the pipette and bath solution (Bao et al., 2004); subsequent work with Panx blocking drugs and siRNA add additional support (Xiao et al., 2012). Positive feedback does not lead to constitutively open channels, however, because ATP binds to the first extracellular loop of Panx1 at residue R75 and forces the channel into a lower, ATP impermeable, sub-conductance state (Qiu and Dahl, 2009). The concentration of ATP needed to stimulate P2X_7_R is 50–100 fold lower than is required to deactivate Panx1 (Qiu et al., 2012), so the system provides an elegant mechanism for amplifying an ATP stimulus while still maintaining control. UTP and GTP will also bind to Panx1 (Ma et al., 2009), but are probably not physiologically relevant because they are unlikely to ever reach sufficient extracellular concentrations to become inhibitory. As should be expected, ATP is not exclusively autocrine in nature, and if Panx1 channels are activated in a single cell, the released nucleotides will diffuse outward to stimulate other receptive cells nearby (Iglesias and Spray, 2012). Some controversy has recently arisen with regard to the “ATP-induced ATP release” mechanism, because Panx1/2 double knockout astrocyte cultures still increase extracellular ATP in a CBX-sensitive response to P2X_7_R activation (Bargiotas et al., 2011). Other ATP release mechanisms are known to be independent of Panx1 (Lemaire et al., 2011; Qiu et al., 2011; Islam et al., 2012), so investigators are encouraged to always exercise caution when interpreting changes in extracellular ATP levels.

### Expression and distribution

Northern blotting was used to generate the first Panx expression profile, using commercially available mRNA samples from mouse (Bruzzone et al., 2003) and human (Baranova et al., 2004). This initial work revealed that Panx1 is expressed in a wide variety of tissues (an observation more recently supported with qPCR by Hanstein et al., 2013), Panx2 is largely restricted to the central nervous system, and there is very little Panx3 expression in any adult tissue with the possible exception of skin. Notable discrepancies existed between these two studies, however, such as very high levels of Panx1 in the heart, skeletal muscle, and testis from the human samples vs. nearly undetectable levels in the mouse, and there was surprisingly weak Panx2 expression in human spinal cord compared to extremely high levels in the mouse. As will be discussed in the following sections, the tissue distribution of Panx mRNA has been reassessed since these preliminary surveys, along with a great number of other details as antibodies have become available. Furthermore, evidence will be reviewed that reveals how Panx expression within tissues is often cell type-specific and, in many cases, developmentally regulated.

#### Trafficking

As integral membrane proteins, Panxs are synthesized at the ER (Vanden Abeele et al., 2006; Boassa et al., 2008; Bhalla-Gehi et al., 2010; Iwamoto et al., 2010). Exogenous over-expression experiments suggest that Panx1 and Panx3 can be retained in the ER as functional calcium leak channels (Vanden Abeele et al., 2006; Ishikawa et al., 2011), and depending on the cell type, the fraction of Panx retained inside the cell can be significant (Penuela et al., 2008). In cell types where targeting to the plasma membrane is evident, however, Panx traffics first to the Golgi via Sar1-dependent COPII vesicles (Bhalla-Gehi et al., 2010) and, in the case of Panx1 at least, glycosylation plays a critical role in regulating this movement (Boassa et al., 2007; Penuela et al., 2007). While in the ER, Panx1 is initially N-glycosylated to a high mannose form (GLY1) that can be separated from the un-glycosylated form (GLY0) using SDS-PAGE; the high mannose polysaccharides are then further processed into a complex form (GLY2) in the Golgi (Boassa et al., 2008). Translocation of Panx to the plasma membrane is severely disrupted in the presence of glycosylation inhibiting drugs or if the extracellular asparagines are mutated (Boassa et al., 2007; Penuela et al., 2007, 2008, 2009; Kurtenbach et al., 2013). Once at the cell surface, Panx1 is sequestered in Triton X-100 insoluble lipid rafts (Dvoriantchikova et al., 2006b), and can remain there for hours or days (Penuela et al., 2007; Boassa et al., 2008). When removed from the membrane, they appear to be shuttled off to lysosomes for degradation (Boassa et al., 2007; Gehi et al., 2011). Internalization does not seem to be mediated by any of the standard clathrin, caveolin, or dynamin-dependent endocytic pathways, so it remains unclear how turnover is coordinated (Gehi et al., 2011). Panx2 is an enigma, because it often localizes to small intracellular vesicles (Zappalà et al., 2007; Lai et al., 2009; Bond et al., 2012). Co-localization of Panx2 and the mannose-6-phosphate receptor suggests that the endolysosome may be at least one intracellular target (Wicki-Stordeur et al., 2013), although no overlap was observed between exogenous GFP tagged Panx2 and LysoTracker® or EEA1 in C6 glioma cells (Lai et al., 2009). Panx2 does, nevertheless, traffic to the plasma membrane under certain circumstances (Ambrosi et al., 2010), perhaps in response to de-palmitoylation (Swayne et al., 2010).

#### Bone

Osteogenic cell types are of particular interest because they are among the very few to express Panx3. Cultured osteoblasts up-regulate Panx3 when stimulated to differentiate and mineralize (Penuela et al., 2007, 2008; Bond et al., 2011; Ishikawa et al., 2011), as do the ATDC5 and N1511 chondrocytic cell lines (Iwamoto et al., 2010). At the tissue level, immunohistochemistry reveals strong Panx3 expression in bones derived from both endochondral ossification (EO) as well as intramembranous ossification (IO) (Wang et al., 2009; Iwamoto et al., 2010; Bond et al., 2011; Ishikawa et al., 2011), and closer examination of EO-derived long bones reveals that growth plate chondrocytes begin to express Panx3 during the pre-hypertrophic to hypertrophic stage of differentiation (Iwamoto et al., 2010; Bond et al., 2011). The transcription factor Runx2 is a key regulator of the *Panx3* gene during osteogenesis (Bond et al., 2011), and over-/under-expression studies have shown how cultured osteoblasts and chondrocytes require Panx3 for normal differentiation (Iwamoto et al., 2010; Ishikawa et al., 2011). Panx1 has also been detected in osteoblasts, although its purpose in these cells has not been explored (Penuela et al., 2008).

#### Bladder

While Panx1 is distributed widely throughout the body, it appears to be particularly enriched in the urinary bladder (Hanstein et al., 2013). Immunohistochemistry reveals substantial expression in the 3–5 layers of urothelial cells closest to the bladder lumen, with little to no expression in the immediately underlying suburothelial layer (Timóteo et al., 2013). Unfortunately, Timóteo et al. did not include the detrusor muscles (responsible for bladder contraction) in their published results, but qPCR indicates that Panx1 can also be found there (Negoro et al., 2013).

#### Brain

RT-PCR amplification has detected mRNA expression of all three rat Panxs in primary astrocytes and whole brain extracts (Lai et al., 2007; Wang et al., 2009). In rodents, relative Panx1 expression within the whole brain peaks in late embryonic/early neonatal animals, and is dramatically reduced in adults (Vogt et al., 2005; Ray et al., 2005). This pattern may arise because neuroblast cells, which are abundant early in brain development, express high levels of Panx1 (Wicki-Stordeur et al., 2012); down-regulation then occurs as these stem cells differentiate into mature neurons and glia. This is not to say that expression is uniformly turned off, as many neural subtypes continue to express appreciable levels of Panx1; these mature neurons include excitatory principal cells, cortical and hippocampal interneurons, GABAergic Purkinje cells, dopaminergic neurons, and cholinergic motoneurons (Ray et al., 2005; Zappalà et al., 2006; Zoidl et al., 2007). *In situ* hybridization and immunofluorescence illustrate this clearly, by most intensely labeling neuron rich regions like the hippocampus (especially the dentate gyrus), cerebellum, inferior olive, substantia nigra, thalamus, layer V of the prefrontal cortex, and the glomular, mitral cell, and granule cell layers of the olfactory bulb (Weickert et al., 2005; Zappalà et al., 2006; Zoidl et al., 2007; Zhang et al., 2012a; Cone et al., 2013). Pools of undifferentiated Panx1-positive neural progenitor cells also remain in the postnatal brain, such as those found in the ependymal/sub-ependymal layer of the lateral ventricles (Wicki-Stordeur et al., 2012; Wicki-Stordeur and Swayne, 2013). At the subcellular level, Panx1 is often observed localizing to neuronal synapses on the postsynaptic side of the cleft (Zoidl et al., 2007), although the soma of certain neurons (particularly in the thalamus) will also sequester Panx1 (Cone et al., 2013). Functionally, it has been proposed that the pool of postsynaptic Panx1 has a role to play in long-term potentiation and synaptic plasticity (Prochnow et al., 2012). While most of the Panx1 expressing cells identified in the brain are positive for NeuN (a neuron specific marker), co-expression with the glial marker GFAP has also been observed in tissue sections (Zappalà et al., 2006; Santiago et al., 2011) and primary astrocyte cultures (Huang et al., 2007; Lai et al., 2007; Locovei et al., 2007; Liu et al., 2008; Iglesias et al., 2009). Cerebellar white matter oligodendrocytes are also a possible source of Panx1 in the brain, but conflicting observations have been reported (Bruzzone et al., 2003; Ray et al., 2005; Vogt et al., 2005; Ray et al., 2006; Zappalà et al., 2006). As will be discussed in more detail below, innate immune system leukocytes express Panx1, and the phagocytic microglia dispersed throughout the brain are no exception (Rigato et al., 2012).

In contrast to Panx1, Panx2 expression is much lower early in brain development, but then becomes more pronounced in the adult (Vogt et al., 2005). The subcellular distribution of Panx2 also changes considerably as neurons mature. It is predominantly intracellular in immature neuronal progenitor cells, then following a brief period where expression is suppressed as the progenitor cells transition toward terminal differentiation, mature neurons shuttle Panx2 all the way to the plasma membrane (Swayne et al., 2010). Many different neural subtypes throughout the brain express the protein, with no apparent correlation to specific neurotransmitter molecules, degree of electrical connectivity, or cellular origin (Zappalà et al., 2007).

Panx3 has been observed at the transcript level in human fetal hippocampal mRNA (Baranova et al., 2004), whole rat brain mRNA (Wang et al., 2009), and cultured rat astrocytes (Lai et al., 2007), but it has been reported as absent from whole mouse brain mRNA (Bruzzone et al., 2003; Penuela et al., 2007; Wang et al., 2009).

#### Circulatory system

Panx1 is found throughout the pulmonary and systemic arterial system, although the relative distribution between the epithelial and smooth muscle layers can be different depending on the artery. For example, Panx1 is readily observed in both the epithelium and smooth muscle of smaller arteries and arterioles, while it is restricted to the endothelium of the aorta and femoral artery (Billaud et al., 2011; Lohman et al., 2012a), and the smooth muscle cells of the middle cerebral artery (Burns et al., 2012). Panx2 can be found in both endothelial and smooth muscle cells of the middle cerebral artery (Burns et al., 2012), and Panx3 has been reported in small arteries less than 100 μm in diameter, particularly in the kidney (Lohman et al., 2012a). Panx1 expression has also been reported in human erythrocytes (Locovei et al., 2006a), although a more recent attempt to detect the protein in these cells with different antibodies was unsuccessful (Melhorn et al., 2013).

#### Ear

Panx1 is expressed by many of the cells found throughout the spiral limbus, organ of Corti, spiral prominence (along the cochlear lateral wall), and Reissner's membrane (Wang et al., 2009). In the organ of Corti, expression is restricted to the rostral epithelial cells early in development (E16.5 in mouse); as the animal matures, expression is induced in both the inner and outer sulcus, Claudius cells, and neurons in the Scarpa's and spiral ganglia (Tang et al., 2008). Panx2 expression has very little overlap with Panx1, except in the Scarpa's and spiral ganglia neurons in the organ of Corti (Tang et al., 2008). There is, however, prominent Panx2 expression in the stria vasularis side of the boundary between the stria vasularis and spiral ligament in the cochlear lateral wall (Wang et al., 2009). Panx3 is restricted to the bones of the cochlear lateral wall and modiolus (Wang et al., 2009).

#### Eye

In the mouse retina, Panx1 is expressed in the ganglion cell layer (along with Panx2), inner nuclear layer, outer plexiform layer, and at the periphery of the outer nuclear layer (primarily in ganglion, amacrine, bipolar, and horizontal cells), with very little expression in the inner plexiform layer. Panx1 levels are highest in the juvenile retina, although retinal ganglion cells retain expression into adulthood (Ray et al., 2005; Dvoriantchikova et al., 2006a,b; Kranz et al., 2013). At the subcellular level, Panx1 can be observed in the dendrites and axons of horizontal cells and type 3a OFF biopolar cells (Kranz et al., 2013). The two Panx1 ohnologs in teleost fish are both represented in the retina with a similar *combined* expression pattern as mammalian Panx1, although each is expressed in different cell layers (Panx1a: outer plexiform layer. Panx1b: inner nuclear and ganglion cell layers) (Prochnow et al., 2009b; Kurtenbach et al., 2013).

#### Gastrointestinal

An immunohistochemistry survey for Panx1 in the human colon (using a polyclonal antibody against a C-terminal peptide) has revealed expression throughout all major layers, with particularly high levels in the submucosal and myenteric ganglia (Diezmos et al., 2013). Gulbransen et al. (2012) have shown that the ganglion neurons, as opposed to supporting glial cells, are the source of this Panx1 expression, and that these neurons are sensitive to extracellular ATP; they undergo apoptosis following activation of P2X_7_R during the early stages of inflammatory bowel diseases (e.g., Crohn's disease). Based on the protective effects of probenecid and ^10^Panx1, it is argued that Panx1 propagates a wave of damage away from the initial point of injury by further increasing extracellular ATP around affected neurons (Gulbransen et al., 2012). It will be important to repeat the work of Gulbransen et al. with Panx1^−/−^ animals, however, because probenecid and ^10^Panx1 have recently been shown to dampen the inflammatory response independent of their effect on Panx1 (Wang et al., 2013a).

#### Immune system

As we will see in section Inflammation and immunity, a lot of effort has been expended to understand how Panxs affect the immune response. The presence of Panx1 in macrophages (including microglia) is well-established (Pelegrin and Surprenant, 2006; Kanneganti et al., 2007; Pelegrin and Surprenant, 2007; Marina-Garcia et al., 2008; Pelegrin et al., 2008; Brough et al., 2009; Lamkanfi et al., 2009; Kronlage et al., 2010; Bargiotas et al., 2011; Lemaire et al., 2011; Qu et al., 2011; Hanley et al., 2012; Rigato et al., 2012); its presence has also been reported in T-cells (Schenk et al., 2008; Woehrle et al., 2010a,b) and polymorphonuclear neutrophils (Chen et al., 2010; Bao et al., 2013).

#### Kidney

Modest expression of Panx1 mRNA and protein has been observed in kidney using Northern blotting (Baranova et al., 2004), qPCR (Ray et al., 2005), and Western blotting (Penuela et al., 2007), while the only support for Panx2 expression comes from a Northern blot screen (Bruzzone et al., 2003). A more recent immunofluorescence survey has revealed the cellular distribution of Panx1 within the nephron, where it can be found in both cortical and medullary structures, particularly on luminal surfaces. The loop of Henle, proximal tubules, and collecting ducts all react to a Panx1 antibody, while the glomerulus and distal convoluted tubules do not (Hanner et al., 2012). In the renal blood vessels, Panx1 appears to be restricted to smooth muscle myocytes, and Panx3 can be found in the juxtaglomerular apparatus and cortical arterioles (Hanner et al., 2012; Lohman et al., 2012a).

#### Lung

Panx1 and a small amount of Panx2 are present in primary airway epithelial (PAE) cells. When these PAE cells are grown in an air-liquid interface culture, Panx1 has a striking subcellular localization to the ciliated apical pole, which is equivalent to the luminal epithelial surface (Ransford et al., 2009). If bathed in hypotonic solution (to induce swelling), PAE cells become much more permeable to propidium iodide and release elevated levels of ATP, and both of these properties are sensitive to Panx inhibitors and Panx1 siRNA (Ransford et al., 2009). Furthermore, trachea explant cultures taken from Panx1^−/−^ mice lose nearly all of their ability to release ATP when exposed to hypotonic solution (Seminario-Vidal et al., 2011), and *Xenopus* lung tissue does not increase ATP release in response to hydrostatic pressure if pre-treated with probenecid (Richter et al., 2014).

#### Reproductive tissue

Both Panx1 and Panx3 are expressed by subsets of cells within the testis, efferent ducts, and epididymis of the male reproductive tract (Turmel et al., 2011). Orchidectomy has little effect on the expression of Panx1 throughout the epididymis, but induces a dramatic up-regulation of Panx3 unless post-operative testosterone is supplied, implying that androgens are able to modulate Panx3 expression (Turmel et al., 2011).

#### Skeletal and cardiac muscle

High levels of Panx1 were observed in human skeletal muscle and heart samples during the initial Northern blot studies performed by Baranova et al. (2004). Further work has since shown that Panx1 levels are actually relatively low in the heart, and that the protein exists primarily in the un-glycosylated state (Kienitz et al., 2011; Dolmatova et al., 2012). That said, Panx1 expression and glycosylation increases significantly if the heart is subjected to an ischemic insult (Dolmatova et al., 2012) or if the myocytes are put into culture (Kienitz et al., 2011). Expression in cultured cardiac myocytes is, however, down-regulated again after a few days (Kienitz et al., 2011). Large (300 pS) probenecid sensitive ion channels correlate with the Panx1 expression in cultured cardiac myocytes, and activation of these channels is sufficient to induce an action potential via voltage-gated sodium channels (Kienitz et al., 2011). The mouse atrial myocyte cell line HL-1 does not normally express Panx1, but it does produce Panx2, and Panx2 siRNA is sufficient to significantly reduce the stretch-activated release of ATP by these cells (Oishi et al., 2012). Subjecting HL-1 cells to hypoxia replicates the up-regulation of Panx1 observed in ischemic heart, and conditioned media harvested from hypoxic cells is able to stimulate a probenecid sensitive fibroblast-to-myofibroblast transition; this is reminiscent of the fibrosis that occurs in the heart during wound healing (Dolmatova et al., 2012). In skeletal muscle, Panx1 channels localize to the luminal surface of T-tubules (Jorquera et al., 2013), where the Panx1-dependent release of ATP is an important mediator of muscle potentiation after repetitive stimuli (Riquelme et al., 2013). Panx1 may also play an ATP-dependent role in maintaining [Ca^2+^]_i_ in muscle fibers after a strong activating stimulus, possibly through an interaction with the dihydropyridine receptor at the sarcoplasmic reticulum (Buvinic et al., 2009; Cea et al., 2012). Over-activity of Panx1, however, is thought to induce myocyte cell death; mutations in the dystrophin gene can up-regulate Panx1 expression, possibly contributing to the pathological apoptosis that leads to muscle wasting in those suffering from muscular dystrophy (Valladares et al., 2013). Similarly, denervated muscles experience significant Panx1 up-regulation along with the onset of atrophy, although the evidence indicates that Cxs have a much larger impact on cell death than Panx1 (Cea et al., 2013).

#### Skin

Immunofluorescence and Western blot studies performed by Celetti et al. (2010) indicate that Panx1 and Panx3 are both present in the epidermis. In mouse skin taken from embryonic stage 13.5, the outermost layer of cuboidal epithelium, as well as the underlying mesenchyme, are highly reactive to Panx1 and Panx3 antibodies. As the mice age, however, epidermal Panx1 expression declines, with what remains being found in the suprabasal cell layer. Forced mis-expression in organotypic culture results in disorganization of the epidermis and reduced vital layer thickness (Celetti et al., 2010). Panx3 is not expressed at appreciable levels in neonatal skin, but diffuse intercellular labeling can be seen throughout the vital layers of adult tissue (Celetti et al., 2010). The primary pool of epidermal Panx3 is found in the sebocytes associated with hair follicles (Celetti et al., 2010; Bond et al., 2011). In human, Western blotting experiments confirm Panx1 expression in subcutaneous fibroblasts (Pinheiro et al., 2013a), and Penuela et al. (2007) have described human Panx1 as being more punctate in confocal images of adult facial epidermis than the mouse homolog. On the other hand, the expression pattern of Panx3 seems to be quite similar between mouse and human (Penuela et al., 2007). Most of the epidermis is comprised of keratinocytes, but melanocytes are also interspersed, and although not explicitly observed *in situ*, a melanocyte cell line has been shown to express Panx1 (Penuela et al., 2012).

#### Tongue

In the large circumvallate papillae (taste buds) found near the base of the tongue, Panx1 is expressed by chemoreceptor (type II) cells, as well as by about 50% of presynaptic (type III) cells. Neither of the other Panx isotypes are present, and only low levels of Panx1 are seen in the surrounding epithelium and muscle (Huang et al., 2007; Romanov et al., 2007).

### Physiological and pathological relevance of pannexins

Pharmacological agents, over-expression techniques, RNAi knockdown, and mouse knockout lines have all been used to modulate the activity of Panx channels both *in vitro* and *in vivo*, in an effort to decipher their functional significance. At least four separate Panx1 knockout mice have now been generated, and all of them are fertile with no outwardly obvious phenotype (Anselmi et al., 2008; Bargiotas et al., 2011; Lemaire et al., 2011; Qu et al., 2011; Santiago et al., 2011; Seminario-Vidal et al., 2011; Dvoriantchikova et al., 2012; Hanley et al., 2012; Romanov et al., 2012; Suadicani et al., 2012). A Panx2 knockout mouse has also been described as viable and healthy (Bargiotas et al., 2011). The relative innocuousness of Panx knockout has actually caused some confusion in the community, as it seems to be at odds with the large body of pharmacological data that has otherwise implied that Panx activity is quite important for a range of biological processes. The underlying cause of these discrepancies is still being sorted out, but they could indicate that the current crop of “specific” Panx inhibitors have off-target effects awaiting identification, or perhaps compensatory mechanisms are up-regulated when Panx is removed from the system. To complicate matters even further, some of the knock-out strategies are not complete; the methodology employed has created hypomorphic tissues instead of completely ablating Panx1 (Hanstein et al., 2013). Irrespective of these caveats, strongly supported cases remain that implicate Panxs in physiological and/or pathological processes, particularly in cells or tissues under stress.

#### Ischemia

The first indication that Panxs might be involved in a specific pathology was observed in acute brain slices, when Roger Thompson and colleagues proposed that the massive disruption in electrochemical gradient which occurs across the plasma membrane of hippocampal neurons challenged with ischemia (i.e., oxygen/glucose deprivation, such as occurs during stroke) could be the result of Panx1 channel activity (Thompson et al., 2006). The depolarization in question does not occur until after about 10 min of continuous ischemic insult, and is reversible if normoxic conditions are reasserted soon after the drop in membrane resistance develops, but it becomes cytotoxic if allowed to persist for more than ~5 min. Panx1 channel blockers, siRNAs, and knockout all lessen damage following oxygen/glucose deprivation, thus supporting the hypothesis that Panx1 is indeed involved (Domercq et al., 2010; Bargiotas et al., 2011; Dvoriantchikova et al., 2012; Jiang et al., 2012). N-methyl-D-aspartate (NMDA) receptors could be the upstream cause of these destructive anoxic depolarization events, by regulating Panx1 permeability via Src family kinase mediated intracellular signaling (Weilinger et al., 2012). In addition to the acute neurotoxic properties of Panx during ischemia, these channels are also thought to have a negative impact during the secondary inflammatory response that follows reperfusion, and this exacerbates damage during the recovery period (Orellana et al., 2011). As discussed in section Brain, Panx1 and Panx2 are both expressed to varying degrees in neurons and supporting glia, and knocking out either gene individually in mice has no significant neuroprotective effects following stroke (Bargiotas et al., 2011). Knocking out both, on the other hand, synergistically reduces total infarct size and functional disability, implying that Panx1 and Panx2 play redundant or compensatory roles in the CNS (Bargiotas et al., 2011, 2012). Similarly, treating mice with probenecid before or after middle cerebral artery occlusion (a common stroke model) reduces infarct volume by 20–30% (Xiong et al., 2014). Another interesting observation during this probenecid study of Xiong et al. was a marked reduction in serum levels of the proinflammatory cytokine HMGB1 in the treatment group. HMGB1 release has also been linked to Panx1 activity following the phenomenon known as “cortical spreading depression,” which precedes migraine headache (Karatas et al., 2013).

In sharp contrast to the detrimental effects Panx expression seems to have in the nervous system during ischemia, Panx-dependent release of ATP in the heart has been identified as a possible component of the cardio-protective phenomena known as ischemic pre-conditioning and post-conditioning. Using the Langendorf *ex vivo* perfused model, Donald Vessey has shown how CBX, mefloquine, and Brilliant Blue G (P2X_7_R inhibitor) will all completely block the cardio-protection gained from ischemic pre-/post-conditioning in a 40 min ischemic insult/40 min reperfusion protocol. These blockers have no effect if exogenous sphingosine 1-phosphate (S1P) or adenosine are present, indicating that release of S1P and/or adenosine is critical (Vessey et al., 2010, 2011a). Furthermore, the short ischemic conditioning events can be replaced by treating the heart with ATP before or after the ischemic insult, but the protective effects of ATP are lost if Panx or P2X_7_R inhibitors are present during the challenge (Vessey et al., 2011b). These effects are likely the result of Akt/PI3K pathway activation, which is primed during pre-conditioning for rapid response to subsequent G-protein coupled receptor activation by S1P (Vessey et al., 2011a,b). Unfortunately, hearts from Panx1^−/−^ animals have not been used to confirm the pharmacological data, so it is still possible that the cardio-protective release of ATP is attributable to some other CBX/mefloquine sensitive mechanism.

#### Seizure

Epileptic-like seizures are caused by dysregulated rhythmic cortical activity. Given that Panx1 can facilitate neuronal depolarization and over-excitability (as discussed in the previous section), it stands to reason that it may also exacerbate, or even cause, these events. To induce epileptic-like seizure in a controlled environment, brain slices can be stimulated with NMDA, or live animals can be treated with the muscarinic acetylcholine 1 receptor agonist pilocarpine or the kainate receptor agonist kainic acid (Thompson et al., 2008; Kim and Kang, 2011; Santiago et al., 2011). Intriguingly, interfering with Panx1 activity (using drugs, RNAi, or knockout) significantly reduces the negative effects of NMDA and kainic acid induced seizures (Thompson et al., 2008; Santiago et al., 2011) while worsening the outcome following pilocarpine treatment (Kim and Kang, 2011). This discrepancy can perhaps be traced back to the different mechanisms leading to hyper-excitability following activation of each receptor type: the muscarinic acetylcholine 1 receptor leads to intracellular accumulation of IP_3_, and the accompanying rise in [Ca^2+^]_i_ has a significant impact on hyper-excitability by depolarizing the cell (Nash et al., 2004), as well as inducing Panx1 opening (Locovei et al., 2006b). If IP_3_ then exits the cell through Panx channels, its intracellular concentration can once again decline, and normal [Ca^2+^]_i_ can be re-established. ATP is released at the same time, and activation of P2X_7_R desensitizes the muscarinic acetylcholine 1 receptor (Fukushi, 1999), so Panx1 and P2X_7_R can ultimately have a quieting effect if pilocarpine is the agonist used to induce seizure. Alternatively, Panx1 opening does not provide negative feedback on the system when NMDA receptors or kainate receptors are the source of epileptic discharge. Over-activation of either receptor will induce sustained status epilepticus; this causes an elevated [K^+^]_*o*_ (Fröhlich et al., 2008), which opens Panx1 channels, thus enhancing excitability by depolarizing the cell and releasing intracellular ATP stores. While P2X_7_R activation may have a quieting effect on the muscarinic acetylcholine 1 receptor, it is believed to potentiate seizure progression when this receptor is not involved, and depleting intracellular ATP stores interferes with the phosphorylation state of GABA receptors to decrease the hyperpolarizing effects of these channels (Whittington et al., 1995). This is a fascinating insight into just how important context can be when interpreting results relating to Panx activity—and, indeed, biological processes in general.

While Panx activity influences the progression of seizure events, the seizures themselves may also influence the expression of Panx. For example, acute mouse hippocampal slices treated with Co^2+^ (another method of evoking seizure events) experience an ~1.5-fold increase in Panx1 and Panx2 mRNA, which does not occur if the actual seizures are suppressed by co-treatment with tetrodotoxin (Mylvaganam et al., 2010). Furthermore, elevated Panx1 expression in cortical layer I has been correlated with human epilepsy, following an analysis of brain tissue collected during temporal lobe resection (Jiang et al., 2013). Whether this up-regulation improves or worsens the pathology, however, is yet to be determined.

#### Calcium waves

Calcium waves occur when a small subset of cells within a population experience a rise in [Ca^2+^]_*i*_, and a signal is then communicated to neighboring cells, which in turn experience their own rise in [Ca^2+^]_*i*_ (Cornell-Bell et al., 1990). This process builds a wave front that can propagate many dozens of cell lengths from its origin, and can even spread between cells not in physical contact, so it is at least partly mediated by an extracellular diffusible agent (Hassinger et al., 1996). ATP has been identified as the primary diffusible agent (Guthrie et al., 1999), which binds to G-protein coupled P2Y receptors on adjacent cells, leading to IP_3_ synthesis and its associated calcium release from the ER. The elevated [Ca^2+^]_i_ ungates Panx1 channels and ATP flows out to propagate the calcium wave onward (Locovei et al., 2006b). The full story is more complicated than this model suggests, however, because gap junctions are also an important mediator of calcium wave progression when cells are in direct contact (Scemes and Giaume, 2006). Furthermore, when Panx1^−/−^ cochlear organotypic cultures were tested for their susceptibility to calcium waves, they did not appear to be hindered (Anselmi et al., 2008). Calcium wave spread may even be exacerbated by Panx1 knockout, at least in cultured astrocytes, possibly as a result of increased Cx43 expression (Suadicani et al., 2012). Given the conflicting reports, further work is required to clarify whether or not Panxs have a significant part to play in physiologically relevant calcium wave propagation.

#### Cancer

Analysis of public gene expression databases reveals a positive correlation between Panx2 and post-diagnostic survival time in glioma patients; these analyses also suggest a link (albeit a much weaker one) between Panx1 and cancer in general (Litvin et al., 2006). Along these lines, over-expression of Panx1 or Panx2 in rat C6 glioma cells has been shown to reduce their tumorigenicity, both *in vitro* and *in vivo* (Lai et al., 2007, 2009). The underlying mechanism for this could reside in a general suppression of cell cycle progression, which has been shown to occur in otherwise normal epidermal keratinocytes over-expressing Panx1 (Celetti et al., 2010), or from enhanced actin/actomyosin facilitated cellular compaction when Panx1 is present (Bao et al., 2012); of course, these two processes are not mutually exclusive. Unlike its effect on glioma, Panx expression in skin cancers may actually be detrimental. Panx1 and Panx3 expression is lower in keratinocyte-derived basal cell carcinoma and squamous cell carcinoma (Cowan et al., 2012), and a correlation has been reported between the aggressiveness of three isogenic metastatic melanoma cell lines and the level of Panx1 expression (Penuela et al., 2012). Knocking down Panx1 from the most aggressive of the melanoma lines suppresses certain oncogenic characteristics, and knock-down also diminishes tumor size and metastases when the cells are implanted onto the chorioallantoic membrane of a chicken embryo (Penuela et al., 2012). From a therapeutic standpoint, Panx1 has been linked to the proinflammatory release of ATP from tumor cells treated with immunogenic cell death (ICD) inducing agents like anthracyclines and oxaliplatin (Wang et al., 2013c; Martins et al., 2014). Unlike the standard model of ATP diffusion through the Panx1 channel, release from cells undergoing ICD occurs through a vesicular process known as “lysosomal exocytosis.” Caspase activity, LAMP1, and Panx1 are all necessary for fusion of ATP filled vesicles with the plasma membrane, but it is currently unclear why (Martins et al., 2014).

#### Cell cycle

If Panxs play a role in tumor growth (see previous section), it is natural to question how these proteins influence cell division. This is one of the rare cases where Panx3 has received some attention, and over-expression experiments indicate that this channel protein is anti-mitotic (Celetti et al., 2010; Iwamoto et al., 2010; Ishikawa et al., 2011). At the biochemical level, two mechanisms have been proposed for these observations:

1. Panx3 expression reduces intracellular cyclic-AMP synthesis in response to growth hormone stimulation, leading to reduced cyclic-AMP-responsive element binding (CREB) protein associated (i.e., pro-mitotic) transcription (Iwamoto et al., 2010).
2. Panx3 expression increases [Ca^2+^]_i_, possibly as an ER leak channel, thus increasing calmodulin activity and calcineurin induced NFATc1 de-phosphorylation, with NFATc1 then promoting differentiation (Ishikawa et al., 2011).

Panx activity is not universally growth suppressive, however, because over-expressing Panx1 in the neuroblastoma N2a cell line has been reported to *double* its proliferation rate (Wicki-Stordeur et al., 2012). The downstream signaling events controlling Panx1 induced N2a replication have not been directly explored yet, but extracellular purines are a known mitogen of neural progenitor cells (Lin et al., 2007).

#### Cellular morphology and motility

Evidence exists that Panx1 (and, to a lesser extent, Panx2) can alter cellular morphology. For example, over-expression in cultured C6 glioma cells has a flattening effect that increases the surface area each cell occupies on the substrate (Lai et al., 2007, 2009). In the case of Panx1, this could be related to interactions between the C-terminal tail of the channel, filamentous actin, and the actin nucleating protein Arp3 (Bhalla-Gehi et al., 2010; Wicki-Stordeur and Swayne, 2013). If Panx1 is able to facilitate nucleation and/or stabilization of actin fibers, then its presence along the entirety of the plasma membrane would prevent formation of leading and trailing edges; in essence, uniform actin polymerization is analogous to the cell trying to move in all directions at once, which simply results in cell spread. Indeed, Panx1 over-expressing C6 cells do tend to have a more developed actin cytoskeleton (Bao et al., 2012). Other incoming signals must still be considered though, because highly motile neuroblast cells express significant levels of Panx1 (Wicki-Stordeur and Swayne, 2013). Presumably the actin nucleating effects of Panx1 at the trailing edge of neuroblast cells are outweighed by depolymerization factors, or Panx1 is disproportionately recruited to the leading edge. Panxs have also been observed to influence the development of neurites in cultured neural cells; the outgrowth of neurites is enhanced in PC12 cells (Lai, 2010) and hampered in N2a cells (Wicki-Stordeur and Swayne, 2013). In both cases, differentiation of the cultured cells is also affected by changes in Panx expression, so it remains unclear whether a *direct* link exists between neuritogenesis and Panx channels.

#### Inflammation and immunity

The induced innate immune response is a crucial line of defense against microorganisms that breach the skin or mucosal epithelial lining of the airway or gut. In very minor cases, where small numbers of foreign invaders are present in otherwise healthy tissue, phagocytes (i.e., macrophages, granulocytes, and dendritic cells) will simply engulf and digest the offending cells or particles. In more severe cases, when the infection is large or associated with cellular distress, cytokines and chemokines are released to organize a more aggressive immune response and inflammation. Two separate activation signals are required within a few hours to activate induced innate immunity: First, incoming cytokine stimulation (e.g., soluble TNF-α) or exposure to a foreign microbe-associated molecular pattern (MAMPs, such as lipopolysaccharides or flagellin) activates the NF-κB pathway, leading to up-regulated production of the inactive “pro” form of IL-1β, as well as production of NLRP3 and inflammasome assembly (Qiao et al., 2012). Pro-IL-1β is stored intracellularly with a half-life of ~2.5 h, or until such time as a second stimulus is encountered, which could be an interaction with an effector T-cell or a “danger signal” like the binding of ATP to P2X receptors (Solle et al., 2001). Following the second signal, inflammasome activation ensues; caspase-1 becomes active, which then processes IL-1β into its bioactive form for release into the surrounding tissue to stimulate inflammation (for reviews please see Schroder and Tschopp, 2010; Takeuchi and Akira, 2010).

**Figure 2:**
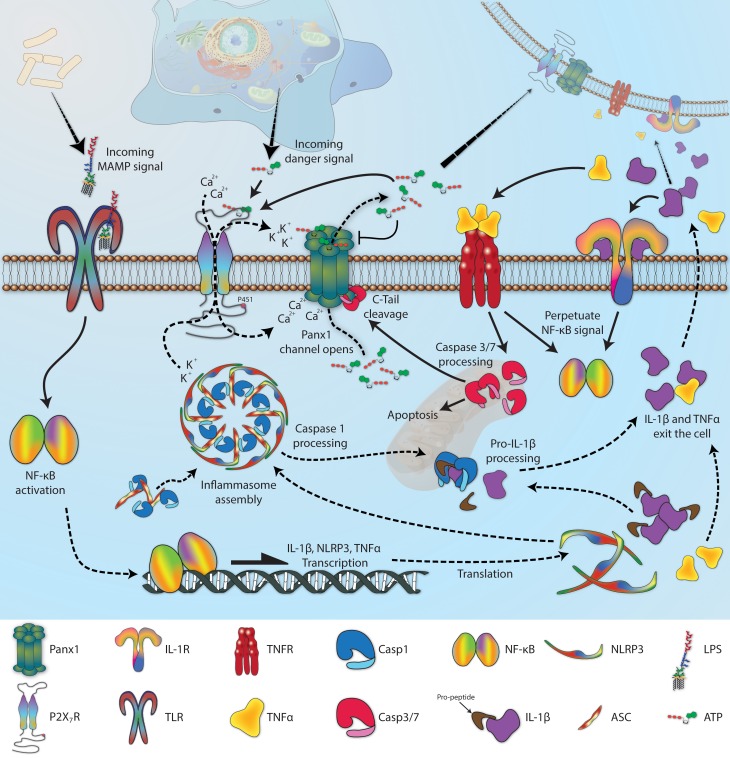
**The role of Panx1 in induced innate immunity**. When an invading microbe breaches an epithelial barrier, microbe-associated molecular patterns (MAMPs) are recognized by phagocytic cells [e.g., lipopolysaccharide (LPS) binding to toll-like receptors (TLR)], leading to an activation of the NF-κ B signaling cascade which up-regulates expression of the inflammasome component NLRP3, pro-IL-1β, and TNFα. Inflammasome assembly brings NLRP3 and pro-caspase1 together, using the adaptor protein ASC, and the whole complex associates with P2X_7_R. If extracellular ATP levels become elevated because of cellular distress in the vicinity (i.e., a “danger signal”), P2X_7_R activation allows calcium to flow into the cell and potassium to flow out-of the cell, along their respective concentration gradients. Lower intracellular potassium is correlated with inflammasome activation, leading to processing of pro-caspase1 into its active state. Panx1 is held in close proximity to P2X_7_R because of an interaction with residue P451 on the C-terminal end of the receptor, allowing local increases in extracellular potassium and intracellular calcium to stimulate channel opening, thus releasing more ATP into the extracellular space. This ATP further stimulates P2X receptors in the surrounding area, but also inhibits Panx1 channels as a negative feedback mechanism, preventing complete collapse of the electrochemical gradient. Meanwhile, liberated caspase1 will cleave the pro-peptide from IL-1β, which is then secreted from the cell along with TNFα. Receptors for both of these cytokines (e.g., IL-1R and TNFR) are also pro-inflammatory, and upon activation will initiate or perpetuate NF-κ B signaling. In addition, TNFR will induce the pro-apoptotic cascade that leads to caspase3 and caspase7 activation; both can clip the C-terminal tail of Panx1, which leads to a constitutively open channel. Several graphics included in this figure were derived from works deposited in the Wikimedia Commons (http://commons.wikimedia.org) by the following authors: LPS, by Mike Jones; DNA, by Sponk; background eukaryotic cell, by Mariana Ruiz; and mitochondria, by Kelvin Song.

Pelegrin and Surprenant (2006) were the first to suggest a connection between Panx1-mediated ATP release and induction of the innate immune response. They drew a link between Panx1 and very low resistance pore formation in macrophages following P2X_7_R stimulation, and speculated that the channel was responsible for inflammasome activation and downstream caspase-1-dependent IL-1β release. A multitude of independent studies followed up on this hypothesis by treating stimulated macrophages with Panx-blocking drugs and/or the ^10^Panx1 mimetic peptide, and both caspase 1 activation and release of IL-1β are indeed suppressed under these conditions (Kanneganti et al., 2007; Pelegrin et al., 2008; Marina-Garcia et al., 2008; Lamkanfi et al., 2009; Wang et al., 2013a). It came as quite a surprise, then, when stimulated Panx1^−/−^ macrophages suffered no impairment of large pore formation, inflammasome assembly/activation, or IL-1β processing/release (Bargiotas et al., 2011; Qu et al., 2011; Turmel et al., 2011; Hanley et al., 2012; Wang et al., 2013a). The reason for this discrepancy is probably an off-target effect of the pharmacological agents (as opposed to a compensatory mechanism induced by loss of Panx1) because the ^10^Panx1 peptide continued to suppress inflammasome activation in Panx1^−/−^ leukocytes (Wang et al., 2013a). Despite the confusion surrounding inflammasome activation, interfering with macrophage Panx1 does cause deficiencies in other respects. For example, probenecid, ^10^Panx1, and Panx1 siRNA all restrict ATP release and NO production in microglia following LPS stimulation (Orellana et al., 2013a). In peritoneal macrophages, cell fusion normally follows cytokine activation, but the process is severely impaired if the leukocytes are harvested from P2X_7_R^−/−^ or Panx1^−/−^ mice (Lemaire et al., 2011). Also, monocyte-recruiting “find-me” signals are released by apoptotic cells when the C-terminal tail of Panx1 channels are cleaved by caspases 3 or caspase 7, so infiltration of infected/damaged tissue by immune cells is slower when Panx1 is removed (Chekeni et al., 2010; Qu et al., 2011). A model that takes into account our current understanding of the induced innate immune response and Panx1 activity is illustrated in Figure 2.

Autoimmunity is an example of inappropriate inflammation becoming pathological, and here too Panx1 has been implicated. Experimental autoimmune encephalomyelitis (EAE) is a common model of multiple sclerosis, which is a neurodegenerative condition that stems from overactive leukocyte diapedesis into the myelinated regions of the CNS. Panx1 expression tends to increase in areas affected by EAE, and Panx1^−/−^ mice experience a delay in symptom onset that correlates well with delayed immune cell infiltration (Lutz et al., 2013; Negoro et al., 2013). It should be noted that the mechanism behind chemotaxis itself is not necessarily dependent on Panx1, because a gradient of complement component 5a is still equally attractive to knockout macrophages (Kronlage et al., 2010).

Macrophages are not the only class of leukocyte functionally modulated by Panx1. In neutrophils, for example, Panx1 appears to be part of a protein complex that includes formyl-peptide receptors, human tweety homolog 3, P2Y_2_R, and actin, that is enriched at the leading edge during mobilization in response to formyl-peptides released from ruptured host cells and/or invading bacteria (Chen et al., 2010). Formyl-peptide induced chemotaxis is completely disrupted if neutrophils are treated with CBX or ^10^Panx1, presumably due to a loss of purinergic signaling cascades at both the leading and trailing edge of the cells (acting through P2Y_2_R and A_2A_ receptors respectively) (Bao et al., 2013). Lymphocytes are also affected by Panx1, such as during times of hypertonic stress when T-cells are activated to form an “immune synapse.” Panx1 co-localizes with P2X_1_R and P2X_4_R at these immune synapses (Woehrle et al., 2010a), and pharmacological blockers of the channel disrupts immune priming (Woehrle et al., 2010b). IL-2 synthesis, calcium intake from the extracellular space, and proliferation in response to T-cell receptor-mediated MAPK activation are all impaired by pharmacological inhibition of Panx1 (Schenk et al., 2008; Woehrle et al., 2010a). To our knowledge, Panx2 and Panx3 expression has not been reported in any type of immune cell, and their absence has been explicitly verified in macrophages (Hanley et al., 2012).

Certain non-immune cell types also modulate immune response during times of stress and, again, correlations with Panx1 expression/activity have been reported. Gingival epithelial cells are the first line of defense in the oral cavity, where ATP stimulated NLRP3 inflammasome activation leads to reactive oxygen (ROS) formation and release; treating these cells with shRNA against P2X_7_R, P2X_4_R, or Panx1 all reduce ROS by 50–75%, and probenecid interferes with IL-1β release (Hung et al., 2013). Lipopolysaccharide or chronic exposure to dietary saturated fatty acids increases expression of panx1 and inflammasome components in hepatocytes, which primes them for IL-1β release and ATP-dependent apoptosis (Csak et al., 2011; Ganz et al., 2011; Xiao et al., 2012). Similarly, neurons are highly sensitive to apoptosis following inflammation, and Panx1 has been linked to neuronal activation of the NLRP1 inflammasome in response to hyperglycemia (Meng et al., 2013) and the AIM inflammasome following exposure to double stranded DNA (Adamczak et al., 2014).

#### Microbial infection

While significant effort has been devoted toward describing how Panxs are involved in the host inflammatory response, a small amount of work has also revealed how Panx can be hijacked by invading pathogens. For example, the severity of erythrocyte lysis following exposure to α-toxins from haemolytic *E. coli* or *S. aureus* is reduced by CBX, mefloquine, or probenecid (Skals et al., 2009, 2011). Assuming these inhibitors are effective due to their ability to block Panx1 channels (the only Panx expressed in red blood cells), then their influence over haemolytic infection could once again relate back to ATP release and activation of a purinergic signaling cascade, because the α-toxin is unable to integrate into the plasma membrane without P2X receptor activation (Skals et al., 2009).

ATP signaling has also been implicated in the early phase of HIV infection, somehow contributing to viral uptake by CD4^+^ host cells (Seror et al., 2011). Panx1 is translocated to areas of contact between viral *env*-containing and CD4/CXCR4-containing membranes, and blocking its activity with mimetic peptide or siRNA substantially decreases the amount of new virus eventually produced by lymphocytes exposed to purified HIV particles (Seror et al., 2011; Orellana et al., 2013b; Paoletti et al., 2013). Common Panx blockers like probenecid and mefloquine are already used to treat human disease (Iglesias et al., 2008; Silverman et al., 2008), so an *in vivo* study is hopefully on the horizon; it will be interesting to see if these drugs have a place in the current anti-retroviral cocktails administered to HIV patients, or perhaps as prophylactics.

#### Response to O_2_ and vascular tone

Following the observation of Panx1 in the membrane of red blood cells, questions were raised about the impact the channel might have on biochemical and autonomic responses to low oxygen (Locovei et al., 2006a). ATP is released from erythrocytes when they encounter an area with reduced O_2_, and this release is sensitive to CBX, probenecid, and the ^10^Panx1 mimetic peptide (Sridharan et al., 2010). An interaction between ATP and P2Y receptors and/or P2X_7_R on the surface of erythrocytes could stimulate even greater movement of ATP out of the cell, along with release of epoxyeicosatrienoic acids (Locovei et al., 2006a; Jiang et al., 2007), leading to activation of receptors on the blood vessel walls. Purines are potent vasodilators when bound to P2Y receptors on vascular endothelium (Burnstock, 2010), so the final result is a widening of blood vessels and greater perfusion of tissue experiencing low oxygen tension (Locovei et al., 2006a). As previously discussed, however, the physiological context that Panx1 finds itself can be an important factor in its downstream effects. ATP is not always vasodilatory, as binding to P2X and P2Y receptors in smooth muscle cells generally causes contraction (Burnstock, 2010). Phenylephrine induced vasoconstriction in the thoracodorsal artery is inhibited by mefloquine, probenecid, and Panx1 siRNA, similar to the effects of blocking P2Y receptors directly (Billaud et al., 2011). It bares repeating, of course, that not all ATP release is dependent on Panx1 channels; in the case of erythrocytes, activation of the β-adrenergic prostacyclin receptor allows ATP to exit the cell regardless of Panx channel activity (Sridharan et al., 2012), while ATP outflow is only cut by about half in Panx1^−/−^ cells (or in wild-type cells treated with probenecid/CBX) following exposure to the wasp venom protein MST7 (Leal Denis et al., 2013).

Panxs may also play a more systemic role in monitoring and responding to blood O_2_ levels. The carotid body is a small structure (near the bifurcation of the carotid artery) that encourages rapid breathing during hypoxia by sending action potentials to the cardiorespiratory centers of the brainstem. Panx1 is expressed in the sustentacular (glomus type-II) cells of the carotid body and is thought to amplify an excitatory ATP signal following P2Y_2_ receptor activation, leading to increased autonomic ventilation rate (Zhang et al., 2012b). Panx1 is also expressed in the glossopharyngeal nerve (GPN) that transmits signals from the carotid body to the brainstem, and it has been proposed that depolarization induced opening of the channels could release ATP, which drives a positive feedback to coordinate activity among the neurons within the fiber (Campanucci et al., 2012).

#### Taste response

In taste buds, the gustatory receptor (type II) cells do not form synapses with the afferent nerves responsible for transmitting information to the brain, but instead are associated with serotonergic presynaptic (type III) cells. ATP is released from tastant activated receptor cells, which then activates P2X_2_R and P2X_3_R on the presynaptic cell to induce neurotransmitter release into the excitatory synapse (Finger et al., 2005). There is currently discord in the literature as to how ATP is released from the receptor cells, with one group claiming Panx1 channels are responsible (Huang et al., 2007; Dando and Roper, 2009; Murata et al., 2010), while another believes that Cx hemichannels are the primary route (Romanov et al., 2007, 2008). Until very recently, both groups relied exclusively on pharmacological methods to support their positions. The evidence initially appeared to favor Panx1, but taste buds harvested from Panx1^−/−^ mice and depolarized by extracellular K^+^ still activated ATP-biosensor cells held in close proximity (Romanov et al., 2012); this work tells us that Panx1 channels are not the only means of ATP release, although it leaves open the possibility of an additive or redundant mechanism. CALHM1 is an ion channel with many structural similarities to Panxs (Siebert et al., 2013) that may play the part of primary ATP channel in type II cells, as cultures taken from Calhm1^−/−^ mice suffer a loss of taste-evoked ATP release (reviewed in Taruno et al., 2013).

#### Vision

Hydrostatic pressure is an important parameter in eye physiology, one that must be tightly controlled to prevent disorders like glaucoma. When the retina experiences a rise in hydrostatic pressure, ATP is released in a CBX-sensitive fashion (Reigada et al., 2008), and the subsequent activation of purinergic receptors can be detrimental to neuronal health (Resta et al., 2007). Such pressure can arise when there is an imbalance between aqueous humor production by the ciliary body and outflow through the trabecular meshwork. Inflow and outflow are both regulated by extracellular ATP, and pharmacological studies implicate Panx1 and Cx43/40 hemichannels as being about equally important in its release from intracellular stores (Li et al., 2010, 2012a). In the lens, hyposmotic stress also causes probenecid-sensitive ATP release, and the downstream signaling leads to elevated Na,K-ATPase activity; the purpose of this effect is presumably to mitigate cell swelling by balancing osmotic driving force (Shahidullah et al., 2012).

In addition to the homeostatic roles Panx1 may play in the eye, it also modulates neuronal activity in the optic tectum (OT) and retina. Using zebrafish larvae, Li et al. (2012b) illustrated how repetitive depolarization of OT neurons stimulates Panx1 channel activity, and the resultant release of chemo-attractants (probably ATP) recruits microglial processes to these over-active cells. Bulbous contacts form between the neural soma and microglial process, and through an ill-defined mechanism, spontaneous and visually evoked neuronal activity is suppressed; put another way, the neuron is encouraged to “take a rest” (Li et al., 2012b). In terms of direct light perception by the retina, Panx1^−/−^ mice experience an elevated response amplitude to light inputs following dark conditioning, thus reducing the resolving power of rod cell receptive fields (Kranz et al., 2013). This inability to see as well in the dark is an excellent example of a phenotype not immediately obvious in a laboratory setting, but would confer a significant disadvantage in the wild.

## Discussion and concluding remarks

More than 13 years have passed since the name “pannexin” was first coined following the revelation that Inxs are represented in chordate lineages, and the subsequent attention directed at these molecules has been very exciting. The channels themselves appear to have largely relinquished their ability to form intercellular gap junctions—a function now performed by the Cxs—and exist instead as large aqueous pores that facilitate communication between the intracellular and extracellular compartments. Significant inroads have been made toward understanding the biophysical properties of these pores, although the field remains splintered over contradictory reports and thirsty for a comprehensive study to reconcile the differences, particularly with respect to channel selectivity and unitary conductance. We also eagerly await the publication of a Panx crystal structure, which would either confirm the collective assumption that Panxs and Cxs are structurally analogous or spark a massive upheaval should something unexpected be observed.

Most cell types express at least one Panx paralog, and the biological impact these channels are claimed to have is as varied and complex as the biological impacts of the ions and molecules they are permeable to. Quite contrary to this seemingly broad importance, however, is the relative innocuousness of systemically blocking activity or completely knocking out Panxs in mice. Does sufficient redundancy exist to compensate when Panxs are removed from the system, and if so, what are those mechanisms? Furthermore, how can such redundancies be retained when evolutionary forces usually conspire to retool or remove biological components with overlapping function? Or perhaps Panx activity is only *really* important during times of stress or disease, so mice in a controlled laboratory setting do not experience fitness deficits if Panx is absent. We must also be cautious of flawed pharmacological inhibitors, which may have led some investigations astray with unforeseen off-target effects. Fortunately, the availability of knockout animals is becoming less of a limiting factor, so these issues will most certainly continue to resolve over the near term.

The pace of Panx research does not appear to be waning and, as we continue to observe these enigmatic channel proteins, they will undoubtedly continue to be implicated in an even greater number of biological processes. One thing to remain mindful of, however, is that the Panxs are not likely to be the “cause” of most of them—at least not in the same sense that enzymes or transcription factors “cause” biological processes. Panxs should be thought of more generally as “enablers,” helping to propagate or amplify signaling cascades across membranes. Assessing the effects of Panx from this perspective could help protect against over-interpretation of experimental outcomes, and will hopefully encourage a more rigorous exploration of alternative or downstream mechanisms.

### Conflict of interest statement

The authors declare that the research was conducted in the absence of any commercial or financial relationships that could be construed as a potential conflict of interest.
